# In Vivo Inhibition of Dipeptidyl Peptidase 4 and Neprilysin Activity Enables Measurement of GLP-1 Secretion in Male Rats

**DOI:** 10.1210/jendso/bvaf173

**Published:** 2025-11-05

**Authors:** Katrine D Galsgaard, Valentina Abba, Rune E Kuhre, Jens J Holst, Bolette Hartmann

**Affiliations:** Department of Biomedical Sciences, Faculty of Health and Medical Sciences, University of Copenhagen, 2200 Copenhagen, Denmark; Department of Biomedical Sciences, Faculty of Health and Medical Sciences, University of Copenhagen, 2200 Copenhagen, Denmark; Department of Biomedical Sciences, Faculty of Health and Medical Sciences, University of Copenhagen, 2200 Copenhagen, Denmark; Diabetes, Obesity & MASH, Research and Development, Novo Nordisk A/S, 2760 Måløv, Denmark; Department of Biomedical Sciences, Faculty of Health and Medical Sciences, University of Copenhagen, 2200 Copenhagen, Denmark; Novo Nordisk Foundation Center for Basic Metabolic Research, Faculty of Health and Medical Sciences, University of Copenhagen, 2200 Copenhagen, Denmark; Department of Biomedical Sciences, Faculty of Health and Medical Sciences, University of Copenhagen, 2200 Copenhagen, Denmark

**Keywords:** dipeptidyl peptidase 4, glucagon-like peptide-1, neprilysin, oral glucose tolerance test, rats

## Abstract

Measuring glucagon-like peptide-1 (GLP-1) in plasma from rodents is difficult since the active peptide GLP-1(7-36)NH_2_ is rapidly degraded into the inactive GLP-1(9-36)NH_2_ by the enzyme dipeptidyl peptidase 4 (DPP-4). Additionally, the endopeptidase neprilysin (NEP24.11) further degrades both forms of GLP-1.

In mice, in vivo inhibition of DPP-4, preferably combined with inhibition of neprilysin, enables measurement of GLP-1 secretion in plasma. We investigated whether the same inhibitions enable measurement of GLP-1 secretion in rats based on quantification of intact GLP-1.

To investigate in vivo degradation of exogenous GLP-1, the neprilysin inhibitor (sacubitril 0.3 mg/kg) and the DPP-4 inhibitor (sitagliptin 10 mg/kg) were injected intravenously 15 minutes before IV administration of GLP-1(7-36)NH_2_ (2 pmol). For endogenous GLP-1, sacubitril and sitagliptin were administered orally to 7-hours fasted rats 60 minutes prior to oral glucose administration (2 g/kg). In both experiments blood was collected, and intact GLP-1 plasma concentrations were measured.

The inhibitors stabilized exogenous GLP-1 and resulted in a 13-fold higher net area under the curve of intact GLP-1 plasma concentrations (509.3 ± 70.62 vs 39.0 ± 21.65 pmol/L*min). The peak of intact GLP-1 concentrations in response to oral glucose was 7-fold higher in inhibitor-treated rats compared to control rats, in which the GLP-1 response was barely detectable.

Thus, in vivo inhibition of both DPP-4 and neprilysin activity stabilized newly secreted GLP-1, thereby enabling estimation of GLP-1 secretion in rats, which is otherwise notoriously difficult due to rapid degradation.

Glucagon-like peptide-1 (GLP-1), secreted from enteroendocrine cells in the gut [1], enhances glucose-stimulated insulin secretion [2] and reduces appetite [3]. These actions have made GLP-1 based drugs highly effective in the treatment of type 2 diabetes [4] and obesity [5]. Upon secretion, the intact or active form of GLP-1, GLP-1(7-36)NH_2_, is almost immediately degraded by the enzyme dipeptidyl peptidase 4 (DPP-4) to GLP-1(9-36)NH_2_ [6, 7], which is mostly inactive. The endopeptidase neprilysin (NEP24.11) further degrades both GLP-1(7-36)NH_2_ and GLP-1(9-36)NH_2_, creating several smaller fragments [8, 9]. Consequently, GLP-1(7-36)NH_2_ is eliminated extremely rapidly from the circulation [10] with a half-life of a few minutes in humans [11, 12], rats [13], and mice [14]. In humans, there is a similar extensive degradation of GLP-1 by DPP-4 [15], but the degradation by neprilysin is less extensive, and it is therefore possible to estimate GLP-1 secretion from measurements of the circulating concentrations of the primary metabolite GLP-1(9-36)amide [16]. As a result, most rodent in vivo studies report minor or no increases in plasma GLP-1 concentrations in response to stimuli that result in robust GLP-1 responses when tested in other models, eg, isolated perfused intestines. Glucose is 1 example of this [17, 18].

Most methods for measurement of GLP-1 are based on high affinity antibodies [ELISA and radioimmunoassays (RIA)] binding to different epitopes of the GLP-1 molecule. Some antibody-based assays apply a “sandwich” method in which 2 antibodies binding to different epitopes are combined [19]. With such assays, the degradation pattern of GLP-1 could lead to a separation of the 2 epitopes and thus no measurable signal. This problem may be circumvented if the assay instead relies on only 1 antibody binding to 1 of the degradation products. However, single antibody assays typically require a large sample volume, making them unapplicable for repeated plasma measurements in rodents, and they are also often less sensitive and more unspecific as they may cross-react with other products from the same pro-peptide as GLP-1 (the major proglucagon fragment) [19, 20]. Additionally, plasma GLP-1 concentrations are in the low picomolar range, which is below the current sensitivity of mass spectrometry-based models. Thus, using a liquid chromatography-tandem mass spectrometry approach, an increase in plasma GLP-1 concentrations after glucose administration could only be detected in individuals with gastrectomy (with a greatly elevated rate of secretion) but not in control subjects [21].

We recently reported that in vivo inhibition of DPP-4 allows measurement of GLP-1 secretion in plasma samples from mice. The best results were obtained when DPP-4 inhibition was combined with inhibition of neprilysin [22]. The pharmacokinetics of GLP-1 are not well described in rats but might be similar to those of mice. The purpose of this study was, therefore, to investigate whether inhibition of DPP-4 and neprilysin enables detection of GLP-1 responses in rats.

## Research Design and Methods

### Animals and Ethical Considerations

All animal studies were approved by the Danish Animal Experiments Inspectorate (2020-15-0201-00756, 2023-15-0201-01409, and 2023-15-0201-01408) and the local ethical committee (Department of Experimental Medicine). Male Wistar rats were obtained from Janvier Labs (Le Genest-Saint-Isle, France). All rats were fed ad libitum with regular chow. Rats were housed 2 to 4 animals per cage in a 12:12-hour light-dark cycle and were allowed at least 1 week of acclimatization prior to experiments.

### In Vivo Degradation of Exogenous GLP-1

Rats weighing approximately 320 g were anesthetized with isoflurane (induction dose 5% and maintenance dose 2-2.5%), and a polyethylene catheter was placed in the right jugular vein. The neprilysin inhibitor sacubitril (0.3 mg/kg body weight, AHU-377; Nordic BioSite, Copenhagen, Denmark) and the DPP-4 inhibitor sitagliptin (10 mg/kg body weight, Januvia; Merck, Sharp & Dohme) were prepared as previously described [22] and dissolved in PBS. The inhibitors were combined and given as a single IV bolus injection through the jugular catheter 15 minutes before administration of GLP-1(7-36)NH_2_ (2 pmol), also administered via the IV catheter. Control rats received PBS instead of the inhibitors. The abdomen was opened by a midline incision and blood samples were drawn from the vena cava 5 minutes before and 10 minutes after inhibitor administration and then 2, 5, 10, and 30 minutes after GLP-1 administration. All blood samples were collected in EDTA coated Eppendorf tubes, which were immediately transferred onto wet ice and centrifuged (6500 rpm, 10 minutes, 4 °C). Plasma was transferred to fresh PCR tubes, which were stored at −20 °C until analysis.

### Oral Glucose Tolerance Test

Rats weighing approximately 300 g were fasted for 7 hours (8 Am to 3 Pm), weighed, and administered sacubitril (0.3 mg/kg body weight) and sitagliptin (10 mg/kg body weight), combined in a single administration, by oral gavage 60 minutes prior to oral glucose administration (2 g/kg body weight). The glucose solution was mixed with acetaminophen (0.1 g/kg body weight) to allow measurement of gastric emptying [23]. Control rats received water mixed with acetaminophen instead of the inhibitors. Blood samples (∼150 µL) were drawn by sublingual vein puncture 5 minutes before and 60 minutes after inhibitor administration and then 5, 10, 30, and 55 minutes after glucose administration.

In a separate study, rats weighing approximately 450 g were not pretreated with inhibitors but received the oral glucose challenge described previously. In these experiments it was possible to collect relatively large blood samples (∼500 µL), allowing measurements with a single site RIA directed at the C-terminus of the molecule. The samples were collected at time point 0, 15, 30, and 60 minutes after glucose challenge and treated as already described.

### Biochemical Measurements

Plasma intact GLP-1 concentrations were measured using an Alpco ELISA kit [Active GLP-1 (7-36)amide Chemiluminescence ELISA (80-GLP1A-CH01), RRID:AB_2941993]. Total GLP-1 plasma concentrations were measured using a Mercodia ELISA kit (catalog no. 10-1278-01, RRID:AB_2892202) or with an in-house RIA (in the experiment with the large blood samples volumes) measuring the sum of total amidated GLP-1 (the sum of 1-36NH_2_, 7-36NH_2_, and 9-36NH_2_), codename 89390 (RRID:AB_2892195) [24]. Plasma acetaminophen concentrations were measured using a spectrophotometric assay (Sekisui, catalog no. Acetaminophen-L3K). Blood glucose was measured with a handheld glucometer (Accu-Chek Mobile U1; F. Hoffmann–La Roche, Basel, Switzerland). Plasma concentrations of insulin and glucagon were measured using the Rat Insulin ELISA (Mercodia, catalog no. 10-1250-01, RRID:AB_2811229) and Glucagon ELISA (Mercodia, catalog no. 10-1281-01, RRID:AB_2783839).

### Statistics

All statistical analyses were done in GraphPad Prism version 10.2.0 (La Jolla, CA, USA). Net area under the curve (_n_AUC) was calculated using the trapezoid rule; baseline was set to the first data point and peaks above and below the baseline were considered. Groups were compared by repeated measurements 1-way or 2-way ANOVA corrected for multiple testing as indicated in the figure legends. *P* < .05 was considered significant. Data are shown as mean ± SEM.

## Results

### In Vivo Inhibition of DPP-4 and Neprilysin Protects Degradation of Exogenous GLP-1

**Figure 1.**
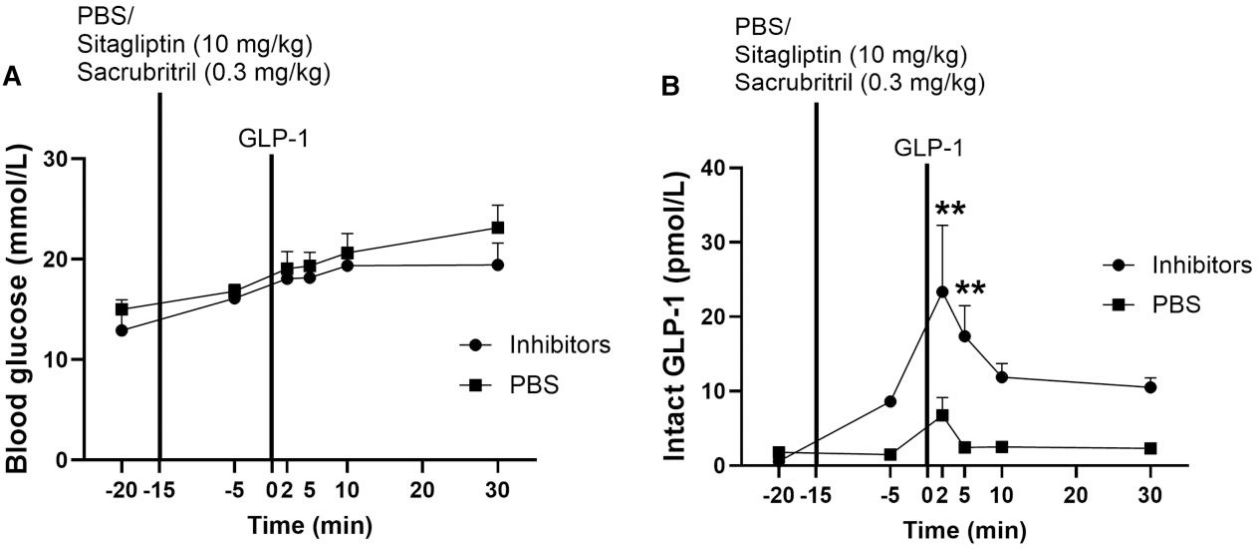
In vivo inhibition of DPP-4 and neprilysin protects degradation of exogenous GLP-1. (A) Blood glucose and (B) intact GLP-1 plasma concentrations (the −5 minutes datapoint of intact GLP-1 from 1 inhibitor-treated rat and 1 PBS-treated rat were missing) in rats treated with DPP-4 inhibitor (sitagliptin) and neprilysin inhibitor (sacubitril) (circle symbols) or PBS (square symbols) prior to GLP-1 (2 pmol) administration. Data shown as mean ± SEM, n = 3-4, *P*-value by mixed-effects analysis corrected for multiple comparisons using Sidak correction. Abbreviations: DPP-4, dipeptidyl peptidase 4; GLP-1, glucagon-like peptide-1.

During the experiment, blood glucose concentrations were similar in inhibitor- and PBS-treated rats (*P* > .8) (Fig. 1A). Consistent with previous reports [25], isoflurane increased blood glucose concentrations. Administration of GLP-1 resulted in robust increases in plasma concentrations of intact GLP-1 in inhibitor-treated rats. In PBS-treated rats, concentrations of intact GLP-1 were generally very low and only elevated at the 2 minutes time-point (Fig. 1B). The GLP-1 peak was almost 3.5-fold higher in the inhibitor group (23.4 ± 8.9 vs 6.8 ± 2.4 pmol/L), and the _n_AUC was 13-fold higher in the inhibitor group (_n_AUC_(-)20min-30min_: 509.3 ± 70.6 vs 39.0 ± 21.7 pmol/L*min, *P* = .008).

### In Vivo Inhibition of DPP-4 and Neprilysin Enables Measurement of GLP-1 Secretion

**Figure 2.**
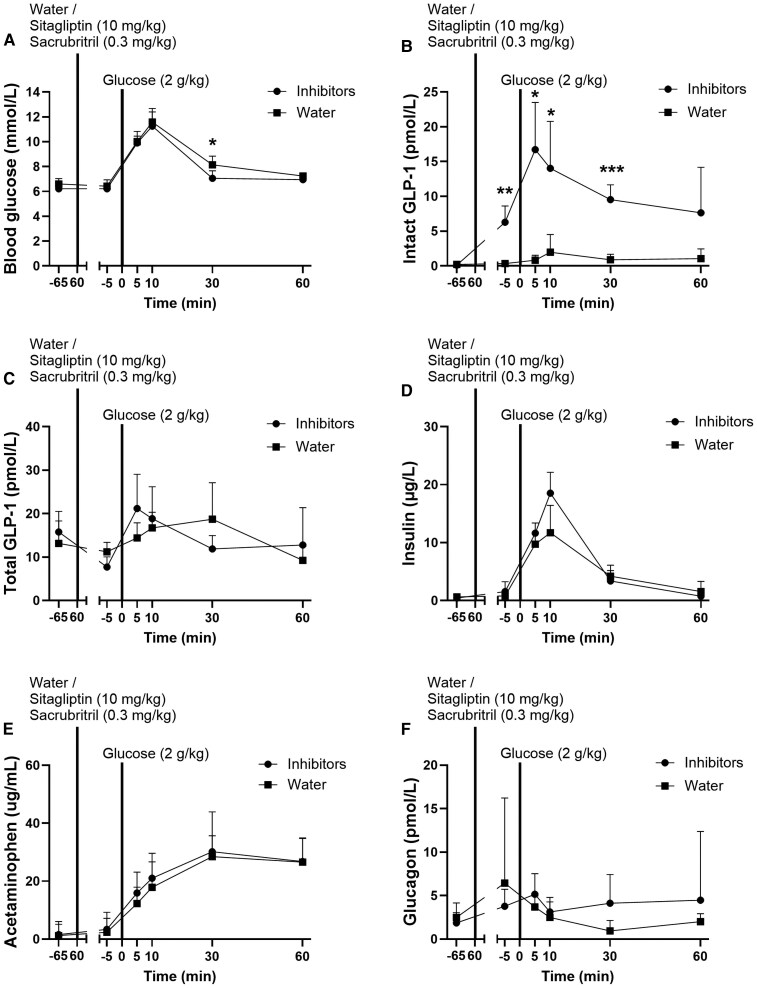
In vivo inhibition of DPP-4 and neprilysin enables measurement of GLP-1 secretion. (A) Blood glucose, (B) intact GLP-1, (C) total GLP-1 (one −5 minutes, two 5 minutes, one 10 minutes, and one 60 minutes datapoint from the inhibitor-treated rats and one −65 minutes and one −5 minutes datapoint from the PBS-treated rats were missing), (D) insulin, (E) acetaminophen (one −65 minutes datapoint was missing from an inhibitor-treated rat), and (F) glucagon plasma concentrations in rats treated with DPP-4 inhibitor (sitagliptin) and neprilysin inhibitor (sacubitril) (circle symbols) or water (square symbols) prior to glucose (2 g/kg) administration. Data shown as mean ± SEM, n = 6, (A, B, C, and F), *P*-value by 2-way ANOVA and (D and E) mixed-effects analysis, both corrected for multiple comparisons using Sidak correction. Abbreviations: DPP-4, dipeptidyl peptidase 4; GLP-1, glucagon-like peptide-1.

Rats with and without prior inhibitor treatment received an oral glucose challenge to stimulate GLP-1 secretion. Thirty minutes after glucose administration, blood glucose concentrations were lower in inhibitor-treated rats compared to water-treated rats (*P* = .04) (Fig. 2A). However, this was not reflected by differences in _n_AUC (_n_AUC_(-)65min-30min_: 125.7 ± 18.9 vs 134.3 ± 12.6 mmol/L*min, *P* = .7). In inhibitor-treated rats, plasma concentrations of intact GLP-1 were significantly increased 55 minutes after the inhibitor treatment and rose further at 5, 10, and 30 minutes after glucose administration (*P* < .02). In the water group, no significant increase was detected (*P* > .3). Thus, intact GLP-1 plasma concentrations were higher in inhibitor-treated rats compared with water-treated rats 55 minutes following the inhibitor treatment and 5, 10, and 30 minutes following glucose administration (*P* < .04) (Fig. 2B). At the 10 minutes time-point, intact GLP-1 concentrations were 7-fold higher in inhibitor-treated rats compared to control rats (14.0 ± 2.8 vs 2.0 ± 1.0 pmol/L, *P* = .03). In contrast, total GLP-1 plasma concentrations were similar between groups (*P* > .1) (Fig. 2C). Insulin concentrations increased in both inhibitor- and water-treated rats (*P* < .0001) and tended to be higher in inhibitor-treated rats when compared with water-treated rats 10 minutes after glucose administration (*P* = .1) (Fig. 2D). Acetaminophen plasma concentrations, a proxy of gastric emptying, were similar between groups (*P* > .9) (Fig. 2E), as were plasma concentrations of glucagon (*P* > .3) (Fig. 2F).

### Concentrations of Endogenous Intact GLP-1 Are Barely Detectable Without Prior DPP-4 and Neprilysin Inhibition

**Figure 3.**
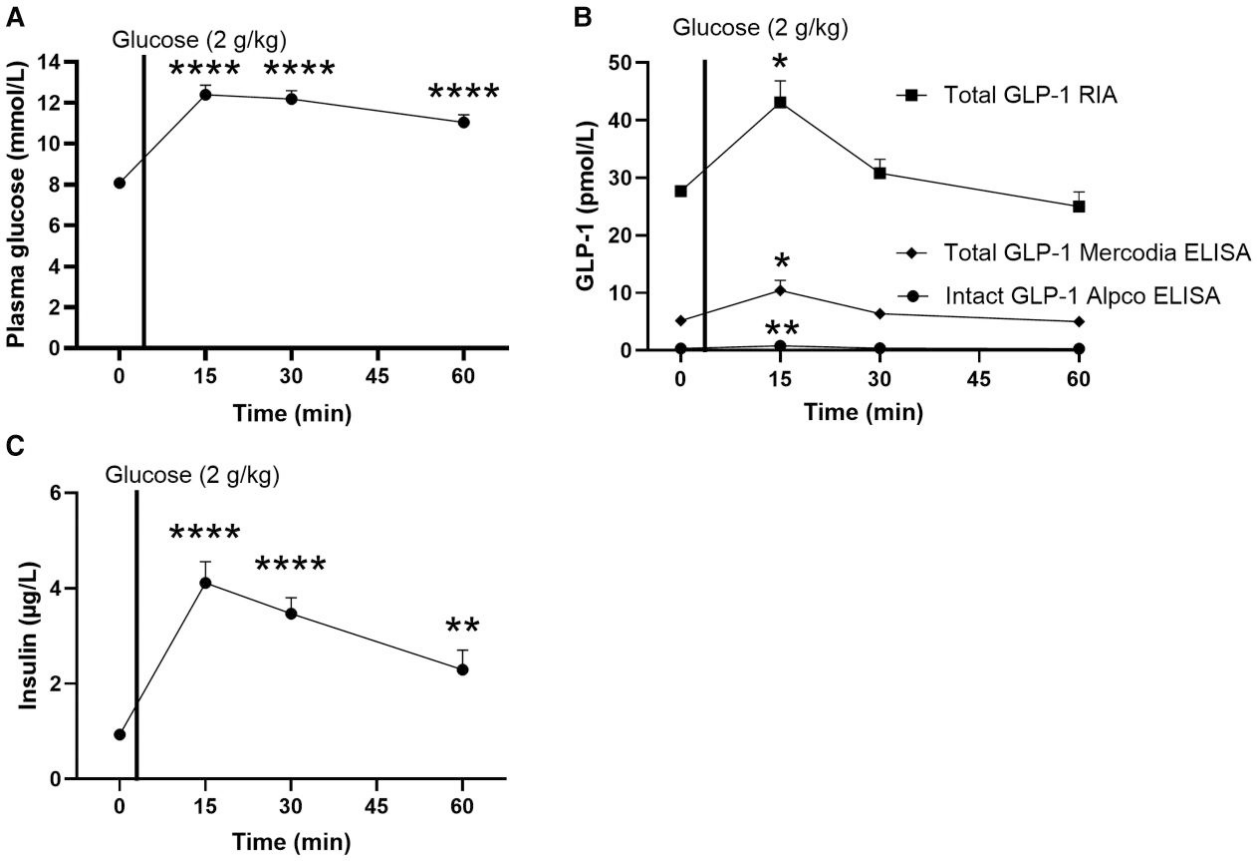
The endogenous secretion of intact GLP-1 is low without prior DPP-4 and neprilysin inhibition. (A) Blood glucose and (B) total GLP-1 concentrations measured using RIA (square symbols) or the Mercodia ELISA (diamond symbols), and intact GLP-1 concentrations measured using the Alpco ELISA kit (circle symbols), and (C) insulin in rats following glucose (2 g/kg) administration. Data shown as mean ± SEM, n = 5-6, *P*-value by repeated measurements 1-way ANOVA corrected for multiple comparisons using Dunnett correction. Abbreviations: DPP-4, dipeptidyl peptidase 4; GLP-1, glucagon-like peptide-1; RIA, radioimmunoassays.

To further illustrate the consequences of lack of inhibitor treatment on results of alternative methods for measurement of GLP-1 secretion, rats with no prior DPP-4 and neprilysin inhibitor treatment received an oral glucose challenge. During the challenge, plasma glucose concentrations increased (*P* < .0001) (Fig. 3A). Baseline concentrations of intact GLP-1 (Fig. 3B) were markedly lower than the concentration of total GLP-1 whether determined with single antibody RIA or sandwich ELISA (total GLP-1 RIA: 27.7 ± 1.0 pmol/L, total GLP-1 Mercodia ELISA: 5.2 ± 0.6 pmol/L, and intact GLP-1 Alpco ELISA: 0.4 ± 0.05 pmol/L). However, both of the assays measuring total GLP-1 and the assay measuring intact GLP-1 concentrations detected increased concentrations 15 minutes after glucose administration (total GLP-1 RIA: 43.1 ± 3.7 pmol/L, *P* < .02; total GLP-1 ELISA: 10.5 ± 1.8 pmol/L, *P* < .02; and intact GLP-1 Alpco ELISA: 0.8 ± 0.07 pmol/L, *P* < .008) (Fig. 3B). Insulin concentrations also increased following the glucose administration (*P* < .009) (Fig. 3C).

## Discussion

Here we aimed to find a method that would allow accurate measurements of GLP-1 secretion in response to glucose in plasma samples from rats. Taken together, our results show that in vivo inhibition of DPP-4 combined with inhibition of neprilysin allows detection of a GLP-1 response, which was almost absent without inhibitors. This is in line with what we have previously reported in mice [22].

The experiment in which exogenous GLP-1 was administered confirmed the short half-life of GLP-1 [7, 11] but importantly also showed that the inhibitors stabilized exogenous GLP-1(7-36)NH_2_. This prompted us to investigate if this was also the case for endogenous GLP-1 secreted in response to an oral glucose challenge. Without the inhibitors, plasma concentrations of intact GLP-1 were generally very low, and a response to the glucose challenge was barely detectable and insignificant. With the inhibitors, plasma concentrations increased even before glucose was administered, showing that the inhibitors also stabilized basally secreted GLP-1, and this was followed by a clear peak in response to the glucose challenge, occurring in parallel with the increases in glucose concentrations (reflecting glucose absorption) and slightly ahead of the insulin peak response. An increase in total GLP-1 plasma concentrations was detected in both groups in response to the glucose challenge; however, the plasma concentrations of intact GLP-1 did not align with the total GLP-1 plasma concentrations. The reason for the lack of correlation may be that the kit measuring total GLP-1 cross-reacts with other forms of GLP-1 and/or cross-reacts with GLP-1 containing proglucagon products from the pancreas. In addition, cross-reacting secreted proglucagon products from the gut or pancreas might have a longer plasma half-life, allowing accumulation of the products. Indeed, the concentrations of total GLP-1 decreased during the period following inhibitor treatment and prior to glucose, perhaps pointing to a pancreatic origin of the reactants since the secretion of glucagon from the pancreas would be expected to be inhibited. At least, the intact GLP-1 ELISA that measures GLP-1(7-36)NH_2_, with no cross-reactivity to GLP-1(9-36)NH_2_ or the glycine extended GLP-1(7-37) gave results that were approximately 10-fold lower than the concentrations of total GLP-1 measured by the Mercodia ELISA and about 60-fold lower than those measured by RIA. Nevertheless, all 3 assays measured an increase in GLP-1 concentrations 15 minutes following the glucose administration; the increase measured with the intact ELISA amounted to about 0.3 pmol/L but to around 5 pmol/L with the Mercodia total ELISA and 15 pmol/L with the C-terminal RIA. Since these increases were not reproduced in the intact ELISA, they are unlikely to represent intact GLP-1. The C-terminal RIA, however, does react with C-terminal fragments of GLP-1 so that results with this assay may reflect the secretion of GLP-1 from the L-cell (but detected as elevations in the concentration of a C-terminal fragment). The elevation measured with the Mercodia ELISA cannot be interpreted from knowledge about its cross-reactions, but the assay must react with some of the fragments of GLP-1.

Our results show that GLP-1 secreted in response to glucose is barely detectable in its intact form without prior in vivo inhibition of DPP-4 and neprilysin and clearly represent an underestimation of the full response, which is captured to a larger degree upon in vivo DPP-4 and neprilysin inhibition. This view is supported by the results of the C-terminal RIA, which actually showed a similar elevation after glucose. However, the problem with that approach is the large plasma volumes required for accurate measurements—volumes that are not normally available.

A limitation to the proposed approach is that DPP-4 and neprilysin are enzymes that degrade several other peptides than GLP-1, which could affect secretion of GLP-1 to some extent either directly or indirectly. These peptides include glucagon, which is degraded by neprilysin [26] and glucose-dependent insulinotropic polypeptide, which is degraded by DDP-4 [27]. Thus, the inhibitors may also affect blood glucose and plasma concentrations of insulin. The underlying reasons for these effects require further investigation but may for insulin be due to the stabilizing effects of the inhibitors on intact glucose-dependent insulinotropic polypeptide and GLP-1, leading to a larger incretin potentiation of glucose-stimulated insulin secretion. The fact that the inhibition changes the circulating levels of intact GLP-1 and other DPP-4- and neprilysin-substrates, such as glucagon, with whatever metabolic consequences this may have, makes the approach best suited for studies of *secretion* of GLP-1. We only tested 1 DPP-4 inhibitor, 1 neprilysin inhibitor, and 1 kit for measuring intact GLP-1 (although this was carefully selected). We assume that other DPP-4 inhibitors and neprilysin inhibitors would have the same effects, but this needs to be tested. The DPP-4 inhibitor used has been extensively characterized and has been used for treatment of type 2 diabetes since 2006 [28]. Finally, we only used male Wistar rats and thus cannot make any conclusions regarding female rats or other strains of rats.

In conclusion, our findings show that without prevention of the degradation of GLP-1 in vivo, GLP-1 responses in the rat will be grossly underestimated or missed. Knowledge regarding the actual release of GLP-1 is essential for evaluation of its physiological importance.

## Data Availability

Some or all datasets generated during and/or analyzed during the current study are not publicly available but are available from the corresponding author on reasonable request.
